# Oligodendroglial p130Cas Is a Target of Fyn Kinase Involved in Process Formation, Cell Migration and Survival

**DOI:** 10.1371/journal.pone.0089423

**Published:** 2014-02-21

**Authors:** Constantin Gonsior, Fabien Binamé, Carsten Frühbeis, Nina M. Bauer, Peter Hoch-Kraft, Heiko J. Luhmann, Jacqueline Trotter, Robin White

**Affiliations:** 1 Department of Biology, Molecular Cell Biology, Johannes Gutenberg-University of Mainz, Mainz, Germany; 2 Institute of Physiology, University Medical Center of the Johannes Gutenberg-University, Mainz, Germany; Aix Marseille University, France

## Abstract

Oligodendrocytes are the myelinating glial cells of the central nervous system. In the course of brain development, oligodendrocyte precursor cells migrate, scan the environment and differentiate into mature oligodendrocytes with multiple cellular processes which recognize and ensheath neuronal axons. During differentiation, oligodendrocytes undergo dramatic morphological changes requiring cytoskeletal rearrangements which need to be tightly regulated. The non-receptor tyrosine kinase Fyn plays a central role in oligodendrocyte differentiation and myelination. In order to improve our understanding of the role of oligodendroglial Fyn kinase, we have identified Fyn targets in these cells. Purification and mass-spectrometric analysis of tyrosine-phosphorylated proteins in response to overexpressed active Fyn in the oligodendrocyte precursor cell line Oli-*neu*, yielded the adaptor molecule p130Cas. We analyzed the function of this Fyn target in oligodendroglial cells and observed that reduction of p130Cas levels by siRNA affects process outgrowth, the thickness of cellular processes and migration behavior of Oli-*neu* cells. Furthermore, long term p130Cas reduction results in decreased cell numbers as a result of increased apoptosis in cultured primary oligodendrocytes. Our data contribute to understanding the molecular events taking place during oligodendrocyte migration and morphological differentiation and have implications for myelin formation.

## Introduction

Oligodendrocytes play a key role in central nervous system (CNS) homeostasis. They myelinate neuronal axons and thereby facilitate saltatory conduction of action potentials and provide trophic support for neurons [1]. During CNS development, oligodendrocyte precursor cells (OPCs) migrate from the subventricular zone towards the white matter where they differentiate into myelin-forming oligodendrocytes. This maturation process is accompanied by increasing complexity of cellular process branching as well as an increased expression of several myelin genes [2]. In order to enwrap and myelinate multiple axonal segments, oligodendrocytes synthesize large amounts of myelin lipids and proteins to form the myelin sheath. It was recently demonstrated that cultured oligodendrocytes determine the molecular composition of membrane sheets even in the absence of neurons and that myelin basic protein (MBP) acts as a molecular sieve facilitating a specific lipid to protein ratio in these sheets [3]. In the presence of axons, myelin synthesis appears to be target-orientated and induced by axonal signals. The Src family non-receptor tyrosine kinase Fyn is a key molecule in the oligodendroglial differentiation and myelination process integrating neuronal signals into oligodendrocyte responses [4] and loss of Fyn activity results in hypomyelination in the CNS [5]. Oligodendroglial Fyn can be activated by an F3-contactin/α6β1 integrin complex binding to axonal L1-CAM as well as laminin from the extracellular matrix surrounding the axon [6], [7]. Neuronal activity increases the amount of cell surface L1-CAM and Fyn activity, stimulating myelin formation [8]. The role of integrins in oligodendrocyte survival and the myelination process has been addressed in several studies. Especially the myelination of small diameter axons appears affected in the absence of β1 integrin signals, which may result from aberrant process growth or branching [9], [10].

p130Cas (crk-associated substrate; also known as breast cancer anti-estrogen resistance 1, BCAR1) is an adaptor protein acting as a crucial effector of integrin signalling [11]. It has previously been shown to be phosphorylated by Src family kinases on tyrosine residues and is involved in signalling events connected with various cellular functions such as the organization of the actin cytoskeleton and cell migration [12], [13]. In cerebellar neurons, p130Cas is important for axon elongation and it has been proposed that its tyrosine phosphorylation translates extracellular signals into cytoskeletal changes [14]. Functions of p130Cas in oligodendrocytes have yet to be described. Here we show that p130Cas is expressed during all stages of oligodendrocyte maturation in culture as well as in the oligodendrocyte precursor cell line Oli-*neu*. In oligodendroglial cells, p130Cas is phosphorylated by Fyn, co-immunoprecipitates with Fyn and co-localizes with Fyn at the leading edge of distal processes. Reduction of p130Cas by siRNA impairs cellular process outgrowth and thickness as well as migration of Oli-*neu* cells. Interestingly, prolonged reduction of p130Cas results in increased apoptosis in primary oligodendrocyte cultures causing a reduction in cell number. Our results demonstrate that oligodendroglial p130Cas contributes to the Fyn signalling pathway and affects morphological changes important for oligodendrocyte differentiation and the myelination process.

## Materials and Methods

### Plasmids, siRNA and Antibodies

Generation of the constitutive active (+) and kinase inactive (−) Fyn constructs has been described before [7], [15].

In order to knock down mouse p130Cas, Smartpool SiGenome siRNA (Thermo Scientific, M-041961-00-0005) was used. Non-silencing siRNA (target sequence 5′-AATTCTCCGAACGTGTCACGT-3′, Qiagen) served as control.

The antibodies against CNP (mouse, clone 11-5B) and α-Tubulin (mouse, DM1a) were purchased from Sigma and used 1∶500 and 1∶5000 on Western blot (WB), respectively. Anti-MOG antibody (mouse, clone 8-18C5) was provided by C. Linington (University of Glasgow) and used 1∶500 on WB. Anti-p130Cas antibody (rabbit, C-20) was purchased from Santa Cruz Biotechnology and used 1∶400 on WB or 1∶50 in immunocytochemistry (ICC). The antibody recognizing active Fyn (SRC pY418) was purchased from Life Technologies and used 1∶500 on WB. The antibody against cleaved caspase 3 (rabbit, Asp175) was purchased from Cell Signaling and used 1∶300 on WB. Anti- Glyceraldehyde 3-phosphate dehydrogenase (GAPDH) antibody (rabbit) was purchased from Bethyl Laboratories and used 1∶3000 on WB. Antibodies against Fyn were purchased from Santa Cruz (rabbit, Fyn3, SC-16, 1∶100 in immunoprecipitation) and from BD Biosciences (mouse, clone 25, 1∶250 on WB or 1∶50 in ICC). The antibody against NG2 (rat) was generated in the Trotter laboratory and used 1∶200 in ICC.

### Ethics Statement

Experiments were performed in accordance with the animal policies of the University of Mainz, approved by the German Federal State of Rheinland Pfalz, in accordance with the European Community Council Directive of November 24, 1986 (86_609_EEC). Great care was taken to prevent the animals from suffering.

### Cells and Transfections

Primary oligodendrocytes were obtained from C57BL/6 mice at embryonic day 14-16 as described previously [16]. The primary cells were grown on poly-L-lysine-coated culture vessels in B27 medium containing 1% (v/v) horse serum (life technologies), 10 ng/ml Platelet-Derived Growth Factor-AA and 5 ng/ml basic Fibroblast Growth Factor (both from tebu-bio). The oligodendroglial cell line Oli-*neu* [17] was grown in Sato medium containing 1.5% (v/v) horse serum, also on poly-L-lysine-coated culture vessels.

Oli-*neu* cells were transfected with plasmids using a Gene Pulser Xcell device (Bio-Rad). 10 µg of plasmid DNA were added to 1.8–2 million cells in culture medium and electroporated at 220 V and 950 microfarads (exponential decay program). A medium change was carried out 16–20 hours following transfection. siRNA transfections were carried out with the Basic Nucleofector Kit for Primary Mammalian Neurons (Lonza) according to the manufacturer’s instructions. 160 pmol siRNA were used with 4 million primary oligodendrocytes or 1 million Oli-*neu* cells, respectively.

### Immunocytochemistry and Microscopy

Cells were fixed with 4% (w/v) paraformaldehyde for 15 min and permeabilized with 0.1% (v/v) Triton X-100 in PBS for 2 min, both at room temperature. Blocking was carried out for 1 hour with 10% (v/v) horse serum in PBS. Primary antibodies were allowed to bind for 1.5 hours and secondary antibodies for 25 min in blocking medium at room temperature. For detection, secondary antibodies (Invitrogen and Dianova) were coupled with Alexa488 (1∶400), Cy3 (1∶1000) or Cy5 (1∶100). To stain for filamentous actin (F-actin), phalloidin-TRITC (1∶1000, Sigma) was added during the secondary antibody incubation step. Nuclei were stained with DAPI or Hoechst 33258 (Sigma) for 2 min. Mounting of the cells was carried out using Mowiol.

Images were acquired using a Leica DM 6000 B microscope with a 40x/0.7NA objective lens or a 63x/1.32NA oil objective lens connected to a digital camera (DFC 360) and using LASAF software. Images were adjusted utilizing Adobe Photoshop.

### Cell Lysis and Western Blotting

Cells were harvested by scraping in cold lysis buffer (50 mM Tris pH 7.4, 150 mM NaCl, 1 mM EDTA pH 7.4, 1% (v/v) Nonidet P-40, 0.25% (w/v) sodium deoxycholate) containing HALT protease and phosphatase inhibitors (Thermo Scientific) and incubating on a rotating wheel at 4°C for 45 min. The lysate was cleared from nuclei and debris by centrifugation at 300×g and 4°C for 10 min.

Proteins were separated by SDS-PAGE using a Mini PROTEAN 3 system (Bio-Rad) and transferred onto PVDF membranes (Immobilion-P, Millipore) using a Mini TransBlot Electrophoretic Transfer Cell device (Bio-Rad). To block unspecific binding, membranes were incubated with 4% (w/v) milk (Roth) in TBST (0.05 M Tris, 0.15 M NaCl, pH 7.2, 0.1% (v/v) Tween20) for 30 min at room temperature. Primary antibody binding was carried out overnight at 4°C and appropriate secondary antibodies (coupled with horseradish peroxidase, Dianova) were incubated for 30 min at room temperature, both in blocking medium.

### Immunoprecipitation

All incubation steps were carried out on a rotating wheel at 4°C. For immunoprecipitation (IP) of Fyn kinase, cell lysates were first incubated with 20 µl packed Protein A-Sepharose (Amersham Biosciences) for 45 min (preclear). Subsequently, precleared lysates were incubated with rabbit-anti-Fyn antibody bound to 20 µl packed Protein A-Sepharose for 4 hours. Sepharose beads were washed four times with 1 ml lysis buffer each and once with 1 ml PBS. To uncouple the precipitate, the beads were boiled for 5 min at 90°C in 2x sample buffer containing 200 mM dithiothreitol.

IP of tyrosine-phosphorylated proteins was performed using agarose beads coupled with 4G10 antibody according to the manufacturer’s instructions (Millipore). For mass spectrometric analysis immunoprecipitated tyrosine phosphorylated proteins eluted with sample buffer (2 subsequent elutions) were separated by SDS-PAGE on 4–12% NuPAGE Bis-Tris gels (Life technologies) and stained with Coomassie brilliant blue. By this elution method, the coupled anti-phosphotyrosine antibodies are also removed from the beads and mix with the tyrosine phosphorylated proteins in the eluate. To distinguish antibody subunits (light and heavy chain) from precipitated proteins, as a control the antibody alone was incubated in sample buffer and included on the gel as a control. Excised bands were subjected to trypsin digestion and mass spectrometric analysis.

### Spreading and Process Thickness Analysis

Oli-*neu* cells treated with control or p130Cas-directed siRNA were trypsinized after 24 hours and seeded in serum-free medium. After 30 min (spreading assay) or 4 hours (process thickness analysis), cells were fixed and stained for nuclei to identify single cells and F-actin to outline the cell shape.

In spreading analysis, three distinct types of cell morphology were defined: not spread cells bearing no lamellipodia (cells bearing only filopodia were counted as not spread), cells with lamellipodia (i.e. cells bearing at least one lamellipodium) and cells with lamella (i.e. cells with at least one lamella surrounding the cell on 160°). The percentage of the different shapes was obtained by analyzing 60 cells in each independent experiment.

Process thickness analysis was performed by measuring the width of the thickest process per cell analyzed. Cell processes observed after 4 hours of spreading were similar to neurites: thick processes emerging from the cell body and bearing thinner protrusions such as filopodia. Measurement was performed in a portion of the process in a distance of 6.5 to 13 µm from the nucleus. The maximum width measured in this window was used to report the width of the cell process. The thickest process of 30 cells per condition and the ratio (width siRNA p130Cas)/(width siRNA control) were measured in each independent experiment.

### Migration Assay

Migration analysis was carried out using Boyden chambers (8 µm pore size filter, BD Biosciences) as described before [18]. 2x10^5^ cells were seeded with 200 µl of medium into the upper part and the chamber was placed in a 24-well plate. The bottom well contained 600 µl of medium with 5 ng/ml basic Fibroblast Growth Factor. The cells were allowed to migrate for 6 hours followed by fixation of the membranes with 4% (w/v) paraformaldehyde for 15 min. Non-migrated cells were removed from the top compartment with a cotton swab. Cells that had migrated to the lower side of the filter were stained for nuclei, photographed with an inverted microscope (Leica DM IL with a digital camera DFC 350 FX) and counted.

### TUNEL Assay

For the fluorometric TUNEL assay (DNA Fragmentation Imaging Kit, Roche Applied Science) 5x10^4^ primary mouse oligodendrocytes (1 DIV) were transfected with control or p130Cas siRNA at a final concentration of 80 nM using Lipofectamine RNAiMAX (Invitrogen). 24 h later, control cells were treated with 1 µM staurosporine (Sigma). The relative amount of signal from apoptotic cells (TUNEL-positive) per total amount of cells (nuclear staining) was determined 42 h after transfection.

Oli-*neu* cells (10^6^) were transfected with 160 pmol p130Cas siRNA or control siRNA using the Basic Nucleofector Kit for Primary Neurons (Lonza) according to the manufacturer’s instructions (protocol number O-005). 6 h later control cells were treated with 1 µM staurosporine. 24 h after transfection cells were trypsinized and 1.5×10^4^ cells were seeded in a 96-well culture plate and cultured for 6 h. Fluorometric TUNEL assay was performed as described above.

### Cell Viability (MTT/LDH Assay)

Oli-*neu* cells were transfected with p130Cas siRNA or control siRNA. After 24 h, cells were trypsinized and plated in serum-free medium into 24-well plates (40,000 cells/well). Cell viability was analyzed by the MTT assay 30 min and 6 h after passaging. MTT (0.75 mg/ml 3-(4,5-Dimethylthiazol-2-yl)-2,5-diphenyltetrazolium bromide, Sigma) was added to the medium and cells were incubated for 2 h at 37°C. Formazan crystals formed were solubilized in a buffer containing 40% (v/v) dimethyl-formamide, 10% (w/v) SDS, and 2% (v/v) acetic acid over-night. The absorbance was measured at 570 nm using a plate reader (Tecan Infinite M200). To analyze membrane integrity, cells were subjected to the LDH assay (Cytotoxicity Detection Kit, Roche) 30 min and 6 h after passaging. The LDH assay was carried out as described in the manufacturers protocol with modifications. Cell supernatants were collected and centrifuged at 300 x g before analysis.

## Results

### p130Cas is Associated with and Phosphorylated by Oligodendroglial Fyn Kinase

**Figure 1:**
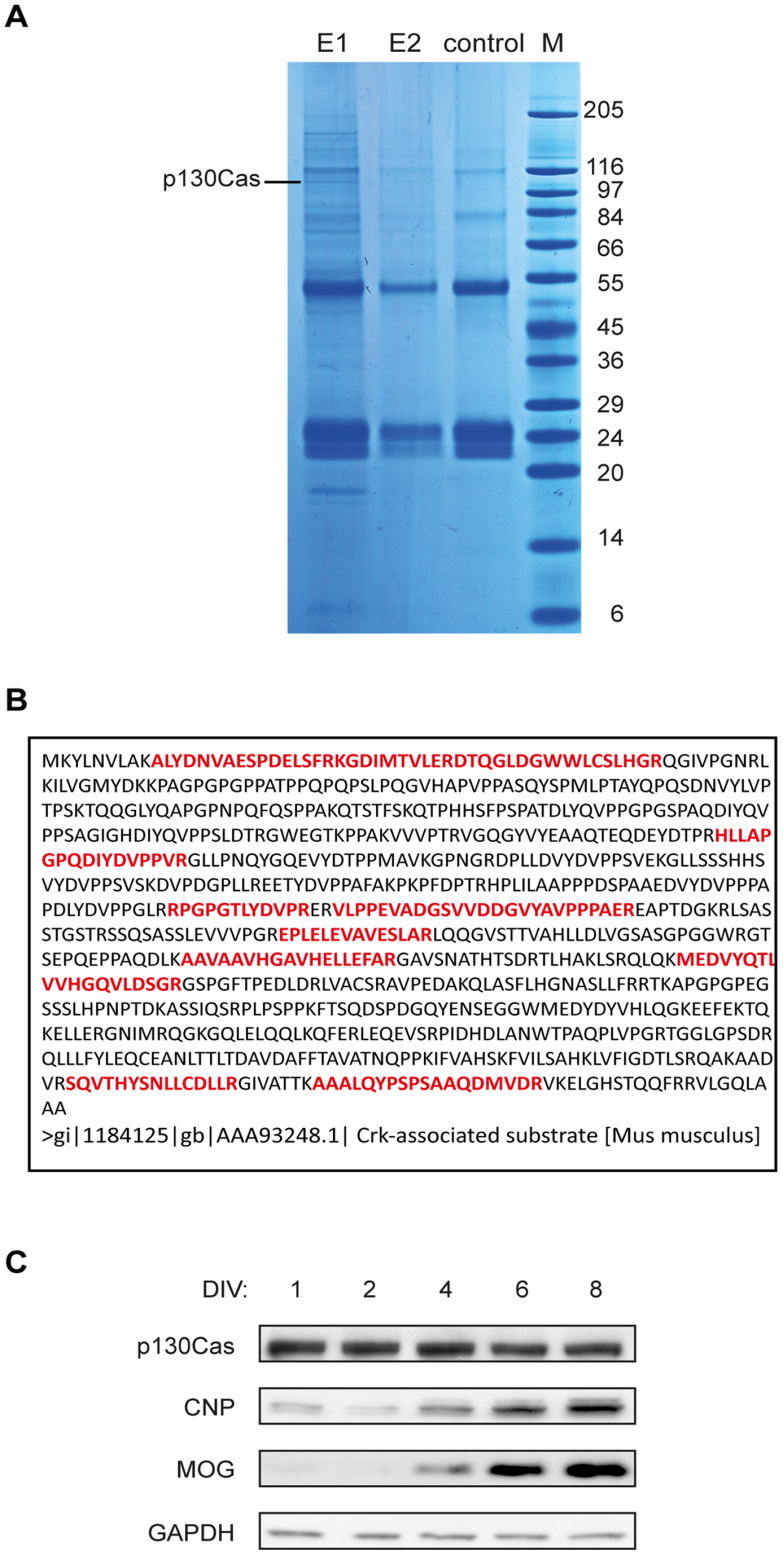
Identification of p130Cas as an oligodendroglial tyrosine-phosphorylated protein in the presence of active Fyn. (A) Tyrosine-phosphorylated proteins were immunoprecipitated from Oli-*neu* cells overexpressing active Fyn. A Coomassie-stained SDS-Polyacrylamide gel with first and second elution (E1 and E2), antibody control (control) and MW marker (M) is shown. The indicated band (p130Cas) was excised and analyzed by mass spectrometry. Molecular weights in kilodalton are indicated on the right. (B) Primary structure of p130Cas. The identified peptides are shown in bold red and cover 20% of the protein sequence. (C) p130Cas expression at different developmental stages in cultured oligodendrocytes (DIV, days in vitro, 1, 2, 4, 6, 8) was analyzed by Western blots with antibodies indicated on the left. CNP and MOG represent early and late maturation markers, respectively, and GAPDH serves as a loading control.

**Figure 2:**
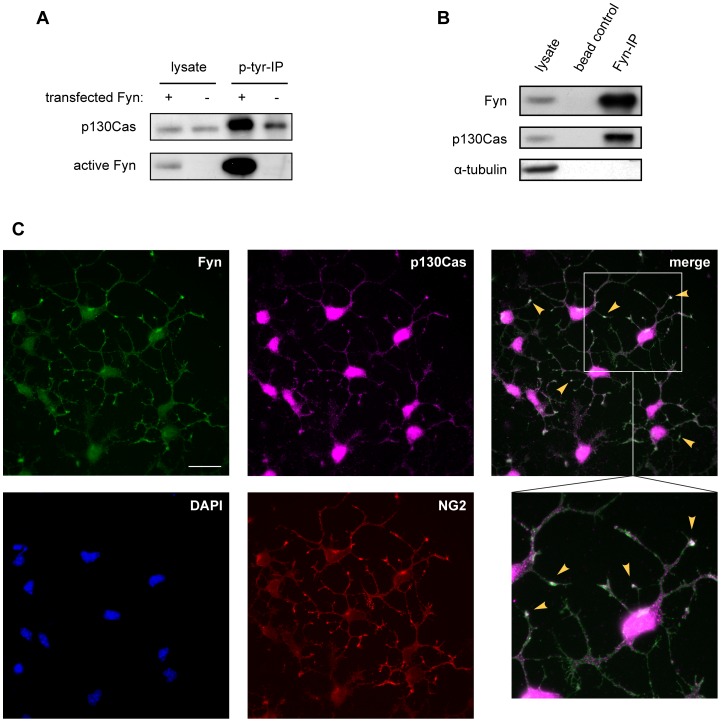
p130Cas is phosphorylated by and associated with Fyn kinase in oligodendroglial cells. (A) Oli-*neu* cells were transfected with expression constructs coding for constitutive active (+) or kinase inactive (−) Fyn, respectively. After 2 days in culture, tyrosine-phosphorylated proteins were immunopurified (p-tyr-IP) from cell lysates and the precipitated amounts were analyzed by Western blotting using antibodies specific for p130Cas and active Fyn kinase. (B) Fyn kinase was immunoprecipitated (Fyn-IP) from Oli-*neu* lysates and the co-purifying p130Cas was identified by Western blotting; α-Tubulin was blotted as control for the specificity of the co-purification. (C) Primary oligodendrocytes were cultured for 2 days and immunostained with antibodies against Fyn, p130Cas and NG2 which is expressed early in oligodendroglial development. Selected areas where Fyn and p130Cas co-localize are highlighted by yellow arrowheads (merge) and shown at higher magnification. Cell nuclei were visualized with DAPI. Scale bar 20 µm.

In a search for oligodendroglial Fyn targets we overexpressed a constitutively active Fyn mutant in Oli-*neu* cells, purified tyrosine phosphorylated proteins and identified them by SDS-PAGE and mass spectrometry (Figure 1A & B). In addition to the previously reported hnRNP F [15], we detected p130Cas. To confirm the expression of p130Cas in oligodendrocytes, primary mouse oligodendrocytes were analyzed by Western blotting and immunocytochemistry using antibodies specific for p130Cas. Figure 1C shows that p130Cas is present in early as well as late developmental stages of primary oligodendrocyte cultures, defined by the levels of the myelin proteins 2′,3′-cyclic nucleotide 3′-phosphodiesterase (CNP) and myelin oligodendrocyte glycoprotein (MOG) which are expressed in more mature oligodendroglial cells. More specifically, p130Cas is localized in the soma and processes in NG2-positive oligodendrocyte precursor cells (Figure 2C), consistent with its previously described involvement in cytoplasmic signaling complexes [13].

p130Cas is a known substrate of Src-family kinases including Fyn in other cell types [13], [14], [19], [20]. To verify that p130Cas is also a target of Fyn kinase in oligodendroglial cells, Oli-*neu* cells were transfected with plasmids expressing either a constitutive active (Fyn+) or a kinase-inactive (Fyn-) form of Fyn. Cell lysates were then prepared, tyrosine-phosphorylated proteins purified by immunoprecipitation and examined for the presence of p130Cas on Western blots. As illustrated in Figure 2A, p130Cas is highly tyrosine-phosphorylated in the presence of active Fyn. This phosphorylation of p130Cas by Fyn requires the interaction of both proteins in a complex. We analyzed this by co-immunoprecipitation as well as co-immunostaining experiments. Fyn was immunoprecipitated from Oli-*neu* cytoplasmic extracts and the precipitated proteins were subjected to Western analysis. Figure 2B depicts that p130Cas co-purifies with Fyn kinase whereas the cytoplasmically localized abundant α-Tubulin is not found in the precipitate. These findings were further substantiated by immunofluorescence stainings of primary oligodendrocytes where p130Cas was found to partially co-localize with Fyn in the cytoplasm, particularly in the tips of the processes (Figure 2C). Taken together, these results suggest that p130Cas is directly phosphorylated by Fyn kinase in the cytoplasm of oligodendrocytes.

### p130Cas is Involved in Process Development and Migration in Oligodendroglial Cells

**Figure 3:**
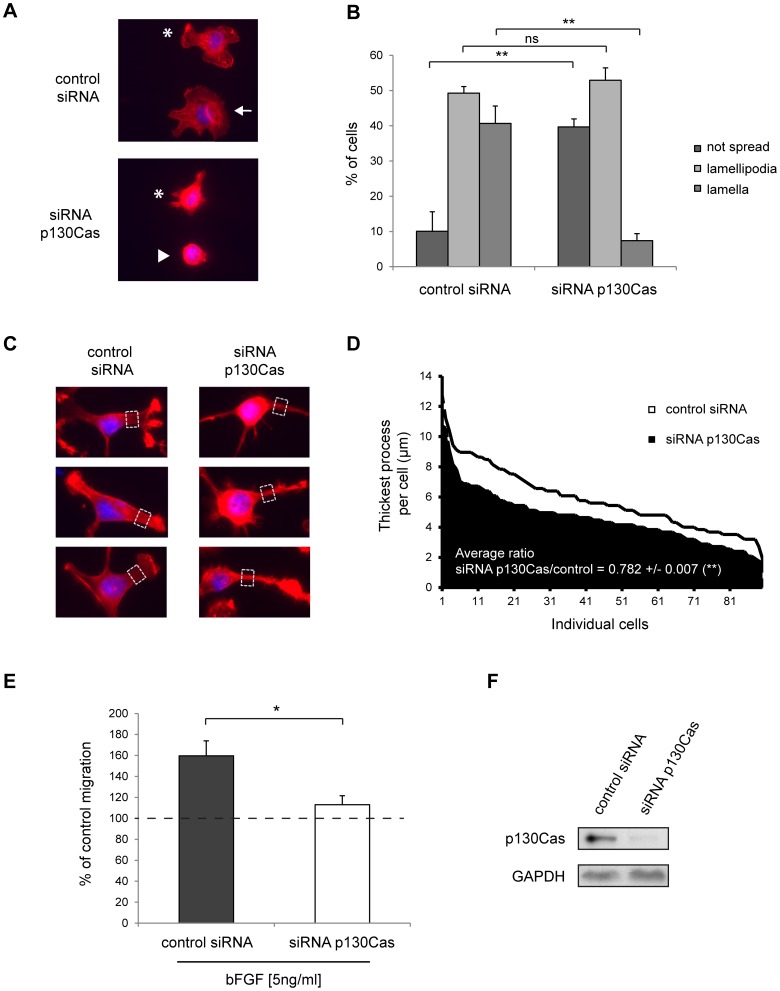
The effect of p130Cas on spreading and migration of oligodendroglial cells. (A) Oli-*neu* cells were treated with p130Cas or control siRNA. 24 hours later, the cells were detached from their culture vessel and 30 min after re-plating they were subjected to immunofluorescence analysis of cell spreading. Cells were classified as either not spread (arrowhead), showing lamellipodia (asterisk) or lamella (arrow). (B) Statistical evaluation of A. Data represents the mean ± SEM from 3 independent experiments; ** p<0.01 (Student’s *t* test). (C) Oli-*neu* cells were treated as in A. Here, immunofluorescence analysis of process thickness was carried out 4 hours after re-plating. Three examples per condition are shown. Areas of measurement are outlined by white dashed rectangles. (D) Statistical evaluation of C. The thickest process for each individual cell was measured and plotted on a chart (from high to low diameters) and the average ratio ± SEM from 3 independent experiments is presented; ** p<0.01 (Student’s *t* test). (E) Oli-*neu* cells were treated with siRNA as in A. After 24 hours, they were re-plated in Boyden chambers and allowed to migrate for 6 hours in the presence of 5 ng/ml bFGF before fixation and migration analysis were carried out. Data are expressed as a percentage of basal migration, i.e. the migration of Oli-*neu* without chemoattractant. Data represent the mean ± SEM from 3 independent experiments; * p<0.05 (Student’s *t* test). (F) Knockdown of p130Cas in Oli-*neu* cells. The siRNA-treated cells as analyzed in A-E were lysed and p130Cas levels were assessed by Western blotting. GAPDH serves as loading control.

Among its diverse functions, p130Cas was shown to participate in actin cytoskeleton remodeling and process outgrowth [14], [21]. To examine whether it has similar functions in oligodendroglial cells, Oli-*neu* cells were treated with siRNA to reduce p130Cas protein levels. After 24 hours, the cells were detached from the cell culture vessel and re-plated, shortly followed by fixation and immunocytochemistry to analyze process formation. Figure 3F shows a Western blot analysis of a fraction of these siRNA-treated cells which confirmed the knockdown of p130Cas when compared to the control siRNA-treated cells. As denoted above, 30 min after re-plating, the cells were fixed and F-actin and cell nuclei were visualized for morphological analysis (Figure 3A). Cells were classified as either not spread, cells with lamellipodia or cells with lamella which represent more mature adhesion sites [22] (for details see Materials and Methods). Compared to control treated cells, reduction of p130Cas levels by siRNA increases the percentage of cells that do not spread out processes and decreases the percentage of cells forming mature lamella (Figure 3B). The percentage of cells forming lamellipodia is not significantly different in cells with reduced p130Cas levels. The overall unaltered percentage of lamellipodia can be explained by the possibility, that although less cells spread out processes to form lamellipodia (which would actually decrease the percentage of cells with lamellipodia), also less cells with lamellipodia develop the more mature lamella. Thus, siRNA-mediated knockdown of p130Cas significantly altered the morphological development of oligodendroglial cells (Figure 3A+B).

Furthermore, we observed an additional difference in oligodendroglial process morphology of p130Cas siRNA-treated compared to control siRNA-treated cells when fixing them 4 hours after plating. F-actin and cell nuclei were stained as stated above. By measuring the diameter of the thickest process in a defined distance from the cell body of individual cells we found that the shape of this newly formed process was affected, with the p130Cas-deficient cells on average exhibiting thinner processes than control cells (Figure 3C+D). In summary, several aspects of (early) oligodendroglial process development seem to be influenced by p130Cas.

In conjunction with its known effects on the actin cytoskeleton and cell spreading, p130Cas has been described as a regulatory factor in migration. Here, the Src-family kinase-dependent tyrosine-phosphorylation of several sites in p130Cas seems to play an important role [23]–[25]. In order to test whether p130Cas also contributes to migration in oligodendrocytes, we performed migration assays using Oli-*neu* cells. 24 hours after treatment with control or p130Cas siRNA, the cells were detached from the culture dish and seeded on top of the porous filter of a Boyden chamber. To stimulate migration of the cells through the pores, bFGF was applied to the lower part of the chamber [26]. After 6 hours of migration, the remaining cells on top of the filter were scraped off. The cells on the lower side of the filter were fixed and their nuclei were stained and counted. Figure 3E illustrates that knockdown of p130Cas levels almost completely abolished bFGF-stimulated migration in oligodendroglial cells.

### p130Cas Reduction Induces Oligodendroglial Apoptosis

**Figure 4:**
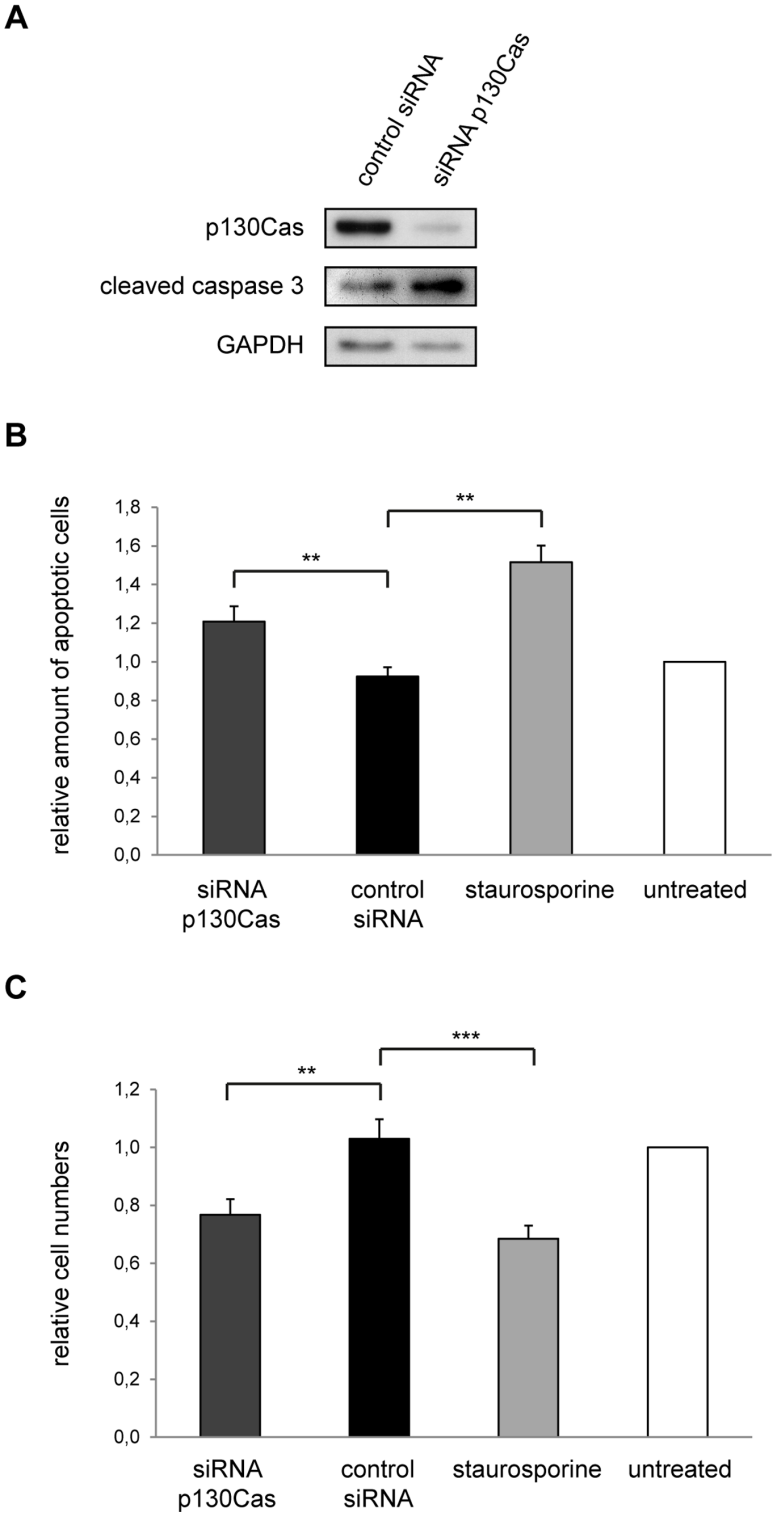
p130Cas is involved in oligodendroglial apoptosis. (A) Primary OPCs were treated with control or p130Cas siRNA. 48 hours later, cells were lysed and tested for levels of p130Cas and cleaved Caspase 3 (indicative of apoptosis) by Western analysis. GAPDH serves as loading control. (B) Primary OPCs were treated with siRNA as in A. Additionally, some cells were treated with the apoptosis inducing agent staurosporine as positive control. After 42 hours, the cells were subjected to a TUNEL assay to test for apoptotic cells. Data show the mean ± SEM from 11 independent experiments; ** p<0.01 (Student’s *t* test). (C) Relative cell numbers from B were counted as additional indication of cell death. Data show the mean ± SEM from 11 independent experiments; ** p<0.01, *** p<0.001 (Student’s *t* test).

p130Cas has been implicated in several pathways influencing cellular survival, particularly as a downstream effector of integrin and growth factor signaling [27]–[29]. We therefore analyzed if reduced p130Cas levels affect oligodendroglial apoptosis. We transfected primary OPCs with control or p130Cas siRNA and performed a Western blot analysis of p130Cas and cleaved Caspase 3 protein levels after 48 hours in culture (2 DIV). As Figure 4A depicts, deficiency in p130Cas resulted in an increased appearance of this early apoptosis marker.

To further validate this finding, a terminal deoxynucleotidyl transferase dUTP nick end labeling (TUNEL) assay was carried out. Primary OPCs were treated with control or p130Cas siRNA as before and cultured for 2 days. Additionally, cells treated with staurosporine, which induces apoptosis, were included in the analysis as a positive control. The labeling of fragmented DNA after 42 hours revealed that a reduction of protein levels of p130Cas leads to increased numbers of apoptotic oligodendrocytes (Figure 4B). Moreover, total cell numbers are significantly decreased by knockdown of p130Cas, resulting from the increased rate of cell death (Figure 4C). Taken together, these results suggest that reduced p130Cas levels cause oligodendroglial apoptosis.

**Figure 5:**
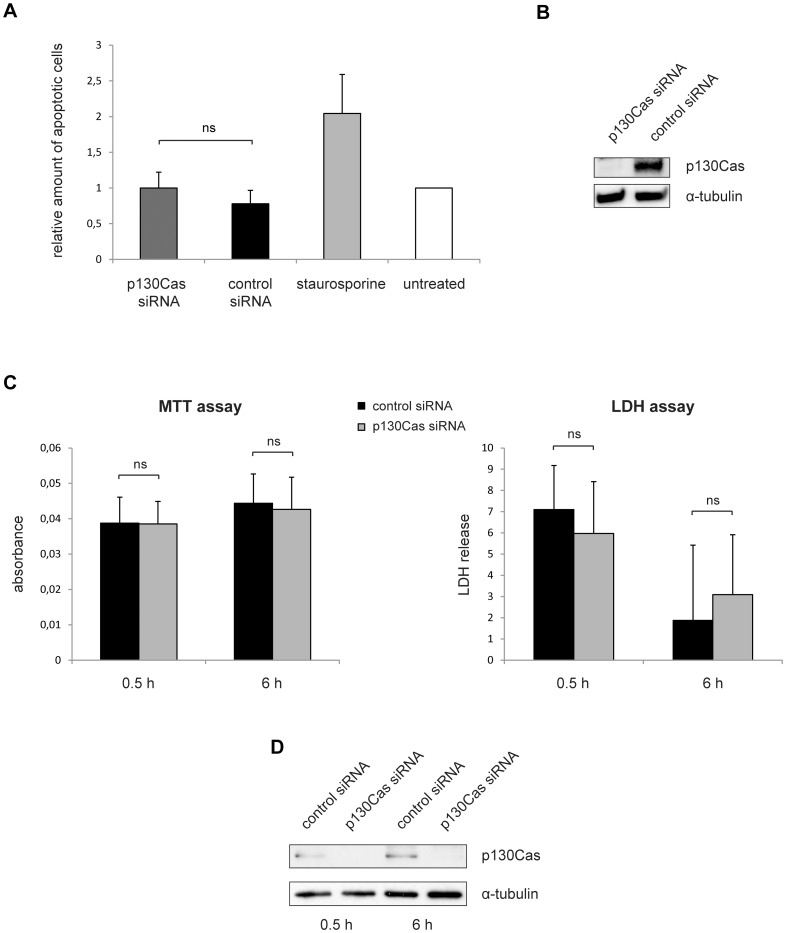
The effect of p130Cas knockdown on spreading and migration in Oli-*neu* does not result from changes in cellular viability. (A) Oli-*neu* cells were treated with p130Cas or control siRNA. 24 hours later, the cells were detached from their culture vessel and 6 h after re-plating a TUNEL assay was performed to test for apoptosis. Data show the mean ± SEM from 3 independent experiments; ns, not significant (Student’s *t* test). (B) Knockdown of p130Cas in Oli-*neu* cells. The siRNA-treated cells as analyzed in A were lysed and p130Cas levels were assessed by Western blotting. α-tubulin serves as loading control. (C) Oli-*neu* cells were treated with p130Cas or control siRNA. 24 h later, the cells were detached from their culture vessel and 0.5 h and 6 h after re-plating MTT and LDH assays were carried out to test for cell viability and membrane integrity, respectively. Data show the mean ± SEM from 3 independent experiments; ns, not significant (Student’s *t* test). (D) Knockdown of p130Cas in Oli-*neu* cells. The siRNA-treated cells as analyzed in C were lysed and p130Cas levels were assessed by Western blotting. α-tubulin serves as loading control.

To assess if our observed p130Cas-mediated effects on process development and migration in Oli-*neu* cells (Figure 3) result from morphological changes caused by apoptosis, we analyzed their cellular viability and apoptotic status under the same experimental conditions. We found that there is no significant difference between Oli-*neu* cells treated with control or p130Cas siRNA with respect to their apoptosis rates (TUNEL assay, Figure 5A) 6 hours after plating which corresponds to the time point used for the migration assay (Figure 3E). In agreement with this, an MTT viability assay (Figure 5C) showed no difference between p130Cas and control siRNA-treated Oli-*neu* cells at the experimental conditions used for the spreading and migration analysis (in Figure 3A,E). In an additional approach we performed an LDH-Cytotoxicity assay and found no significant changes after knockdown of p130Cas in Oli-*neu* cells at the same time points (Figure 5C). We performed Western blots to assess the siRNA-mediated knockdown efficiencies in these experiments and found no detectable p130Cas protein in the p130Cas siRNA-treated cells (Figure 5B+D).

## Discussion

The CNS consists of neurons and glial cells forming an efficient yet extremely complex network of interacting functional units. Specialized membrane extensions of oligodendrocytes elaborate the multilayered myelin sheath in the CNS, a procedure requiring enormous biosynthetic activity. The cells undergo dramatic morphological changes during differentiation *in vivo* which can also be observed *in vitro*. OPCs migrate through the developing CNS and appear to scan the environment for appropriate axonal targets which are recognized and myelinated if certain prerequisites are met [30], [31]. Although a number of signals have been identified which seem to determine the movement of OPCs and the place, timing and rate of myelin formation, a detailed understanding of these mechanisms is still lacking. Myelin synthesis requires complex rearrangements of the oligodendroglial cellular architecture which need to be understood in detail to comprehend the cell biological basics of myelination. The non-receptor Src-family tyrosine kinase Fyn was previously reported as a key signaling component in several cellular processes in oligodendrocytes that are related to the myelination process [4]. We investigated downstream targets of oligodendroglial Fyn kinase and identified p130Cas. In agreement with an analysis of developing mouse brain [14], we showed that p130Cas protein is present at early and late stages of oligodendrocyte differentiation in culture in which the cells express low levels of CNP and no detectable MOG, or high levels of CNP and MOG, respectively. p130Cas is the prototypical member of the Cas family of adaptor proteins which also includes NEDD9 (neural precursor cell expressed, developmentally down-regulated 9), EFS (embryonal Fyn-associated substrate) and CASS4 (Cas scaffolding protein family member 4) [32]. The structure of p130Cas consists of an N-terminal Src-homology 3 (SH3) domain, a proline-rich domain, a substrate domain containing 15 YxxP motifs which are phosphorylated by Src family kinases, a four helix bundle serine-rich domain, and a C-terminal domain containing a bipartite Src binding domain [12]. We confirm here that Fyn interacts with p130Cas as both proteins co-immunoprecipitate and co-localize in Oli-*neu* cells and primary oligodendrocytes. Fyn association with the plasma membrane is determined by myristoylation and additional palmitoylation signals may target Fyn to lipid raft microdomains where Fyn is locally activated by neuronal signals [8], [33]. This suggests that p130Cas and Fyn interact at the plasma membrane and is in agreement with previous findings in which tyrosine phosphorylated p130Cas was shown to be associated with cell membranes in 3Y1 fibroblasts, while unphosphorylated p130Cas is located in the cytoplasm [34]. Furthermore, the Src binding domain was shown to be essential for p130Cas membrane localization [35]. The co-localization appears most prominent in the distal tips of cell processes where tyrosine phosphorylation of p130Cas by Fyn is likely to take place, alluding to a function in focal adhesions and process dynamics.

The adapter molecule p130Cas links extracellular signals with cytoskeletal changes involving the action of integrins and Src family kinases [12], [20], [36], [37] and in lymphocytes and fibroblasts p130Cas is specifically targeted by Fyn [19], [38]. In oligodendrocytes, the roles of integrins and Fyn kinase in cellular differentiation and myelination have been demonstrated in several studies. Both α6β1 integrin and Fyn are involved in the outgrowth of oligodendroglial processes [39]–[41] and myelination [5], [9]. Moreover, p130Cas action on actin cytoskeleton rearrangements was proposed to involve regulation of Rho-family small GTPases [12], [42], [43] and these have also been implicated in oligodendroglial process formation downstream of integrin to Fyn signaling [44]. Compatible with these findings, our data demonstrate a role of oligodendroglial p130Cas in process formation as knockdown of this protein significantly decreases the ability of cells to form lamellipodia and lamella during cell spreading and initial process outgrowth. Furthermore, reduction of p130Cas protein levels appears to affect the lateral growth of processes resulting in significantly thinner processes. Interestingly, lateral growth of oligodendrocyte processes seems to occur during active myelination as has been demonstrated recently in an *in vivo* myelination study in zebrafish [30]. p130Cas has been described to mediate process formation in other cell types including neurons involving the action of Src-family kinases [14] and we propose that this occurs in oligodendrocytes in a similar way. In correlation with the observed morphological changes, reduction of p130Cas also impedes OPC migration. It was shown recently that directional migration of OPCs depends on the action of Rho-family small GTPases activated by the cell surface protein NG2 [18]. As these GTPases have also been suggested as downstream effectors of p130Cas, it is thus likely that it contributes to the above described regulation of OPC migration. In our study, we did not address directly if a Fyn-p130Cas interaction controls migration. However, inhibition of Fyn impedes PDGF-dependent migration in OPCs [45] and it was recently proposed that Slit2 binding to roundabout receptors (Robo) inhibits OPC migration by Fyn inactivation and decreased Fyn/Robo interaction [46]. Moreover, the Src-binding domain and the Src kinase phosphorylation sites in the substrate domain are instrumental in regulating p130Cas-dependent migration [37], [47].

p130Cas knockout mice show cardiovascular abnormalities and die embryonically at day 11.5–12.5 [21]. So far, no oligodendrocyte-specific knockout of p130Cas has been described. In our experiments prolonged knockdown of p130Cas ultimately leads to increased apoptosis levels and reduced OPC numbers in culture (Figure 4). A role of p130Cas in cell death was proposed before [32] and it has been shown in cancer cells that inhibition of Src kinases as well as overexpression of dominant negative p130Cas mutants induce apoptosis [29]. Interestingly it was shown that p130Cas is cleaved by activated Caspase 3 at the onset of apoptosis leading to disassembly of focal adhesion complexes and interfering with survival signaling from the extracellular matrix [48]. Moreover, Fyn kinase plays an important role in integrin-dependent survival signaling in oligodendrocytes [6], [49] suggesting that p130Cas and Fyn contribute to the same pathway in oligodendroglial cells.

We analyzed the viability and apoptotic status of Oli-*neu* cells which were used in our spreading, process thickness and migration assays and did not see any differences between p130Cas siRNA and control siRNA-treated cells under the same experimental conditions. It is thus unlikely that the morphological changes observed in our cell spreading assays and migration phenotype after p130Cas knockdown in Oli-*neu* cells result from the initiation of apoptosis. The apoptosis assay in Oli-*neu* cells was performed 6 h after replating the siRNA-treated cells, because this was the latest time point used in our experiments with Oli-*neu* cells (the migration assay, Figure 3E). As we did not observe a significant induction of apoptosis of p130Cas siRNA-treated cells at that time point, we exclude any apoptotic effects to have occurred earlier during the spreading assay or process thickness analysis, which were performed 30 minutes and 4 h after replating the cells, respectively. Reassuringly, the MTT viability assay and LDH cytotoxicity assay revealed no effect of p130Cas knockdown 30 minutes or 6 h after replating the Oli-*neu* cells. The induction of apoptosis observed in post-mitotic primary oligodendrocytes (Figure 4) was not expected to occur in a comparable manner in the immortalized Oli-*neu* cell line.

In conclusion, we have confirmed expression of the adaptor molecule p130Cas by oligodendrocytes, shown it to be phosphorylated by Fyn kinase and to be involved in oligodendroglial process outgrowth, migration and apoptosis. Our results may stimulate further research regarding the role of p130Cas in the regulation of oligodendrocyte differentiation and survival, as well as myelination or remyelination in pathological conditions.
